# The Hospital. Nursing Section

**Published:** 1906-11-24

**Authors:** 


### The Hospital.

### Nursing Section.

Contributions for "THE HOSPITAL," should be addressed to the EDITOR, "THE HOSPITAL" NURSING SECTION, 28 & 29 Southampton Street, Strand, London, W.C.

No. 1,053.—VOL. XLI. SATURDAY, NOVEMBER 24, 1906.

### Notes on News from the Nursing World.

### OUR CLOTHING DISTRIBUTION.

THE number of parcels delivered at our office marked "Clothing Distribution" is not so great as we could wish, but they are coming in steadily, and with the beginning of the last month of the year next week, we hope that the list of acknowledgments will become longer in every issue up to Christmas. There is one point which we should like to press upon the attention of our readers. It has not the charm of novelty, but it needs to be re-stated. Of course, we prefer, as almoners to the hospitals and infirmaries, large packages of clothing; but we know that the vast majority of our readers have neither time nor money to send more than a single article. We plead, therefore, specially for the supply of single articles, however small or inexpensive, so long as they are useful. The following contributions have reached us since last week: Miss C. Davidson, Holloway; Nurse Farrell, Studley; L. Richardson, Hexham-on-Tyne; Anon., Ilford; and Mrs. Henslowe, Weymouth. Address, The Editor, 28 and 29 Southampton Street, Strand, London, W.C., with "Clothing Distribution" marked outside the parcel.

### THE IMPERIAL MILITARY NURSING SERVICE.

WE are officially informed that Miss E. H. Davies, Miss N. R. McNeil, and Miss K. F. G. Skinner have been appointed staff nurses in Queen Alexandra's Imperial Military Nursing Service. Miss E. M. Pettle, sister, has been transferred to the Military Hospital, Cairo, from the Military Hospital, Valletta. Miss A. C. M. Jameson, staff nurse, has been posted to the Connaught Hospital, Aldershot, on her appointment. The appointments of Miss E. G. Barrett, Miss M. E. Brewer, Miss J. G. Dalton, Miss E. B. Darnell, Miss M. Fisher, and Miss M. German as staff nurses have been confirmed.

### THE MEMORIAL TO ARMY SISTERS.

THE announcement that Lord Selborne and the Governor of Cape Colony last week attended a service in celebration of the resumption of the building of Cape Town Cathedral, will interest many present and late members of the Army Nursing Service and Princess Christian's Army Nursing Reserve. Those who subscribed to the memorial to the sisters who lost their lives during the war, to be placed in the memorial chapel of the Cathedral, will be glad to learn that something is being done towards the completion of the edifice, as up to the present time the sum subscribed has merely been accumulating in a bank.

### CHANGES AT ST. MARY'S HOSPITAL.

A NEW night superintendent has just been appointed at St. Mary's Hospital, Paddington, in succession to Miss Harvey, who held the position for five years and a half. The appointment is interesting as being the first since Miss Davies, the new matron, took up her duties. The new night superintendent was trained at King's College Hospital. Miss Harvey severed her association with the hospital in order to open a nursing home in Norfolk Square. Her resignation has caused much regret, and the staff at St. Mary's wish her success in the arduous undertaking upon which she has entered.

### MISS HUGHES AT READING.

AT the annual meeting of the Queen Victoria Institute for Nursing the Sick Poor of Reading, Mr. M. J. Sutton, who moved the adoption of the report, said that they had had a very favourable year. He mentioned as one of the satisfactory features that, as against twenty-two persons who last year gave something to the Institute as a thanksgiving at the end of their illness, the number in the past twelve months rose to eighty-five; and that these, without any solicitation, contributed altogether £21 7s. 6d. Other encouraging facts were a substantial increase in the annual subscriptions and the interest shown in the movement by the surrounding villages, some of which had signified a desire to share in the work. After several speeches had been delivered Miss Amy Hughes addressed the meeting, and, alluding to the recent formation of the Berkshire County Nursing Association, said that the objects of the County Associations were just as much a part of the Queen's Institute as the training and recommending of nurses. Miss Hughes described the County Associations as the result of the cry of the country districts for women who would take up the work in those lonely districts where there was no hope for Queen's nurses. She saw no reason why the Institute in Reading should not be the centre for the training of nurses for the whole of the country. In proposing a vote of thanks to Miss Hughes, Mr. W. J. Brain stated that the Committee of the Institute were now arranging for one of their staff to continue work in the schools under the Education Authority till the end of the winter, and he thought that it was very probable they would be called upon afterwards to provide a nurse to examine the children in the various schools in the town.

### PROBATIONERS WITHOUT SURGICAL TRAINING.

THE Medical Officer of Exeter Workhouse has reported to the Guardians upon a scheme for the employment and training of probationers in the new Infirmary. He regards it with favour on the ground that it would mean the substitution of a superior class to the wardswoman at present in occupation, and better continuity of work, because the probationers would be required to sign a three years' agreement. He thinks that there is plenty of material available to give young women willing and apt to learn a thorough training for poor-law service. But he candidly affirms that their surgical training will be "somewhat incomplete owing to the fact that all cases for operations are sent to the Exeter Hospital." In order to carry the scheme into effect the staff would have to embrace a superintendent nurse, two charge nurses, four probationers—or six when the old Infirmary has to be used as well as the new—a pauper inmate to do the roughest work, and a male nurse for the male annexe and lunacy ward. The weakness of the scheme, as far as the probationers are concerned, is, of course, the inability to provide for adequate surgical training. Still, its adoption will mark a step in advance, and some day, perhaps, Exeter Poor-law Infirmary may be a fully equipped nurse-training school.

### A NURSES' EXCHANGE IN PRACTICE.

At a Conference in Leeds last week for systematising hospital work in the West Riding of Yorkshire, the subject of the Nurses' Exchange and Central Bureau, was discussed. Dr. Kaye stated that he had taken the matter up with a view to hospital authorities making uniform arrangements with their nurses, and placing them at the disposal of a Central Exchange, so that they might be available for different institutions, as circumstances demanded. A few nurses had been put on the Exchange, and 19 transfers had been made; but the number of nurses had not been equal to the number of applications for them. Dr. Kaye repeated that the essence of his scheme was that the whole of the hospital boards should work in common, and he did not think that the movement would succeed until they did so. It was objected by other speakers that such a system of exchange would interfere with good management, and that the nurses themselves might not like to be banded about from one part to another at a moment's notice. Another objection was that under the present limited operation of the scheme there was a danger of hospital boards entering on the Exchange list only the nurses whom they desired to get rid of. The question is to come up again in January at a larger conference, to which representatives are to be invited from all the county boroughs.

### STRANGE CAUSE OF RESIGNATIONS.

A very unusual investigation has just been concluded by the Irish Local Government Board in connection with the Banbridge Infirmary and Fever Hospital. The facts are, shortly, that the superintendent nurse and three assistant nurses sent in their resignations to the Guardians on the ground that another nurse in the hospital had contracted a contagious disease. It appears that they were afraid of infection, because all the nurses occupied the same sitting-room, their food being cooked and the vessels washed in the same kitchen by the same attendants. The Local Government Board, who have just issued their report, whilst expressing sympathy with the sick nurse, intimate their opinion that she acted imprudently in neither asking for nor accepting leave of absence until she was free from infection. They consider that it would be unfortunate if the Guardians were to permanently lose the services of the superintendent nurse, who, to quote their words, "had so materially assisted in bringing their infirmary and fever hospital up to its present efficient condition," and of the other nurses who seemed to have done their duty faithfully. They therefore urged the Guardians to re-elect them all. The Guardians, however, have since decided, after discussion, to advertise for nurses. We cannot understand how such a position ever arose. Surely, as soon as it was known that the nurse was suffering from a contagious disease, she should have been isolated, and she should never have had any option in the matter. The trouble, if this was the only trouble, would then have been at once at an end.

### THE CHARGE AGAINST AN ATTENDANT.

We notice that the unfortunate woman who was accused at the Hertford Assizes of murdering her husband by administering a noxious drug, and was discharged by the judge, is described in the daily press as a nurse. As a matter of fact, she has only been employed as an attendant in Norfolk. She was never engaged in any capacity whatever at Leavesden Asylum. Her late husband, whom she met at Leavesden, was not an attendant, but he was engineer at that institution.

### THE QUESTION OF TRAINING AT YARMOUTH.

The Yarmouth Guardians appointed at their last meeting a committee of inquiry for the purpose of dealing with the nursing question. It was stated that there has been a marked improvement in recent years. At one time, it is admitted, resignations of nurses were frequent and friction often occurred. But since the practice of promoting probationers has been adopted things are said to have worked much more smoothly. This, however, does not affect the point raised by Dr. Shaw, the medical officer, who maintains that there is a need in the infirmary ward for more fully qualified nurses. It is true that one of the Guardians traverses this statement and affirms that, all the ailments of the patients being senile decay and consumption, the existing staff is equal to their requirements. His colleagues very wisely determined that the opinion of the medical officer ought not lightly to be set aside; and they were also impressed by some useful particulars cited as to the practice in other unions, tending very strongly to prove that, in the interests of both efficiency and economy, there should be more trained and fewer untrained nurses employed.

### PARTIALLY TRAINED PROBATIONERS AT THE ANTIPODES.

At a recent meeting of the Committee of Wallsend Hospital, New South Wales, a discussion took place concerning the terms on which a probationer with some previous experience should be engaged. It was urged that custom was opposed to any distinction as to salary being made between the novice and the partly-trained. Some of the Committee, however, favoured the modification of the rules in relation to the appointment of probationers, but they were in a minority. With regard to the general question, it would obviously be quite impossible for any well-conducted hospital to accept a partially-trained person as probationer on different conditions from an ordinary applicant, because the Australian Trained Nurses' Association only gives its certificate to nurses undergoing a full training in one of the hospitals recognised by them.

### TRAINING HOME IN THE WEST OF ENGLAND.

Mention was made in our columns a few weeks ago of a meeting which was held at Plymouth of a provisional committee formed in order to promote the establishment of a training home for nurses in the three towns of Plymouth, Devonport, and Stonehouse on the lines of the Maternity Home at Plaistow. Lord Mount Edgcumbe, who originated the movement, now states that the development of the scheme will not be hampered at the outset by financial difficulties, two anonymous subscriptions of £100 each having been received, as well as a few other substantial sums and a good many smaller ones. Lord Mount Edgcumbe intimates that the great object in view at present is to extend the area in which the nurses employed by the Devon County Association can find work, even though no pecuniary recognition of it is obtained, in order that a reliable opinion may be formed as to the limits of operations in the future. The immediate need is therefore the increase of the nursing staff. There seems to be every reason to anticipate that the result arrived at—the provision of a convenient centre for training of West Country cottage nurses—will be achieved.

### BEQUESTS TO NURSES.

The remarkable will of Mrs. Ada Lewis-Hill has now been proved, and in addition to a series of splendid legacies to charities, she has left a large number of interesting bequests. The sum of £200 is bequeathed to Nurse Warrington, and of £50 to Nurse Caroline Marsh. If no nursing organisations benefit by the will the nursing staffs of some of the hospitals will indirectly be the gainers, since the amounts assigned to several of the latter will substantially augment their resources.

### PROGRESS AT SHEFFIELD.

At the end of three years the Council of the Sheffield Queen Victoria District Nursing Association have succeeded in putting the organisation on a sound financial basis. At the annual meeting it was announced that the report showed a balance in hand of practically £23; a small amount it is true, but more than enough to justify congratulations, seeing that last year there was a deficit of £204. The welcome change in the situation is partly due to special entertainments on behalf of the fund, and partly to an increase of nearly £100 in the subscriptions. During the twelve months the superintendent and ten nurses paid 34,824 visits, but a considerably larger staff is required to extend the scope of the work. The admirable progress already made goes far to warrant the hope that Sheffield will do its duty in the future.

### CENTRAL MIDWIVES BOARD EXAMINATION.

The number of candidates examined at the examination held by the Central Midwives Board on October 25 shows a marked increase. Of 446, as against 245 on August 1, 346 passed, and the percentage of failures was 22.4, compared with 21.6. Of the successful candidates, Queen Charlotte's Hospital supplied the largest number, 30; but 26 were trained at the Maternity Charity, Plaistow, and 25 at the General Lying-in Hospital. Clapham Maternity Hospital supplied 13 successful candidates; the Rotunda Hospital, Dublin, 12; the London Hospital, 11; the Liverpool Lying-in Hospital, 10; Guy's Institution, seven; the Hospital for Women, Brighton, and Bristol Royal Infirmary, six each; Bristol General Hospital, the Salvation Army Maternity Hospital, and Cardiff Queen's Nurses' Institute, two each. The candidates trained in poor-law institutions were six each at Chorlton Union Hospital, Brownlow Hill Infirmary, Liverpool, and Belfast Union Maternity Hospital; four at Walton Workhouse, Liverpool; three at Birmingham Workhouse Infirmary; two each at Cardiff Union Infirmary and West Ham Union Infirmary, and one each at Sheffield Union Hospital and King's Norton Union Infirmary.

### INCORPORATED SOCIETY OF TRAINED MASSEUSES.

The next examination of the Incorporated Society of Trained Masseuses will be on the following dates: Written examination, Thursday, January 24; Anatomy, and Practical, on Friday and Saturday, February 8 and 9, Monday and Tuesday, February 11 and 12, and Friday and Saturday, February 15 and 16. Forms of application must be filled in and returned to the Secretary not later than January 4. An examination will also be held in Dublin in January and February.

### SHORT ITEMS.

The Duchess of Albany paid an unannounced visit last week to the Hospital for Sick Children, Great Ormond Street, and subsequently went over the Nurses' Home.—An address on the aims and objects of the Royal National Pension Fund for Nurses will be delivered by the Secretary, Mr. Louis H. M. Dick, at the Philosophical Hall, Park Row, Leeds, on Wednesday next at 3.30, when the attendance of all nurses is invited.—The late Miss Mary Isabella Carver, of Southport, has left the sum of £1,000 to the Southport and Birkdale District Nursing Society and Invalid Kitchen; and the late Mr. Richard M. Porritt, of Ramsbottom, the sum of £200 to the Chester-le-Street Nursing Association.—Mrs. Walker, matron of the Small-pox Hospital at Newport, Mon., fell into the canal at the Alexandra Dock the other day, and was drowned, her husband vainly attempting to save her.

### The Nursing Outlook.

"From magnanimity, all fears above;
From nobler recompense, above applause,
Which owes to man's short outlook all its charm."

### THE MIDWIVES BOARD AND POOR-LAW INSTITUTIONS.

Much misapprehension appears to exist as to the bearing of the correspondence which has taken place between the Midwives Board and the Privy Council in regard to the new rules. It may be well therefore to explain the position fully. The act as it stands empowers the Central Midwives Board to frame rules subject to the approval of the Privy Council. The act does not however empower the Privy Council to do anything more than approve or disapprove these rules. The Midwives Board enacted, in their rules, that no person should be admitted to an examination, unless her certificates proved, that, she had undergone a course of training, under supervision satisfactory to the Central Midwives Board. The Privy Council, however, at the instance of the Local Government Board, desire to modify this regulation in favour of candidates from poor-law institutions, by dispensing with the requirement that the supervision of the training shall be satisfactory to the Central Midwives Board. The Privy Council further propose, in the case of candidates from poor-law institutions, that, instead of the certificate, that a candidate has attended a course of instruction in the subjects enumerated in rule D 4, being given in the form prescribed by the Central Midwives Board, and signed by one or more registered medical practitioners recognised by that Board as teachers, such certificate may be signed by the duly appointed chief medical officer of the poor-law institution, or other medical practitioner authorised by the Guardians, with the approval of the Local Government Board, to give such instruction. It will be observed that if the modifications introduced into the new rules, at the instance of the Local Government Board, are to stand, two standards will be set up. One, the higher standard of the Central Midwives Board. The other, that under the less efficient and strict system formulated by the Privy Council, in regard to poor-law institutions, at the instance of the Local Government Board.

It is right that all, who are inclined to criticise the Central Midwives Board, should clearly understand, that, that Board has only been working for three years, and that it had to encounter many difficulties at the outset, owing to the absence of any control or standard in regard to midwives in the past. The Midwives Board is entitled to claim a little more time for developing and bringing into action the important organisation and system, for the improvement of midwives and the protection of their patients, the creation of which has been entrusted to it by Parliament. On the whole the act is working well, though it has not been an easy one to work. We hold, with most reasonable people, that what the Midwives Board needs at the moment, is, the sympathetic support of all who desire to improve the condition of midwives, and a greater freedom from irresponsible criticism, much of which has been based upon imperfect knowledge, or a failure to apprehend both the difficulties and dangers to mothers, attaching to the work of midwives all over the country.

As to the attempt of the Local Government Board to secure for poor-law institutions, freedom from the safeguards which the Midwives Board has wisely set up, in regard to the teaching and theoretical and practical training of candidates for the office of midwife, in connection with midwifery schools, we are of opinion, that the Privy Council must be under a serious misapprehension, as to the hygienic arrangements and circumstances of several of the older and smaller workhouses, throughout the country. If they had had an intimate knowledge of the actual condition and management of many of these institutions, they would never, in our judgment, have consented to urge the Midwives Board to accept the modifications, embodied in the letter from the Privy Council, which was read at a meeting of the Board on July 26th last. The Privy Council's action appears to be based upon the impression, that every workhouse has a paid medical officer, whose duty it is to attend all cases of midwifery; whereas in fact a very limited number of normal cases in workhouses are actually so attended, and midwives as a rule undertake these normal cases. The Midwives Board insists, that thorough medical inspection of poor-law institutions is essential, because they admit general cases of disease, as well as lying-in cases. Now, the actual hygienic supervision exercised by the Local Government Board over poor-law institutions is by means of two medical inspectors, one for London and one for the whole of England and Wales. It follows that the Midwives Board, if it does its duty, must regard all institutions in the light of training schools. This necessity compels them to withhold their approval, where they have evidence of serious structural or administrative defects.

The Midwives Board further disagrees with the acceptance, by the Local Government Board, of a midwife, who has not herself proved her qualifications, as a teacher after examination, to train and instruct pupils in poor-law institutions. How can a midwife, who is certified only as *bona fide*, be expected to train all midwifery pupils efficiently in practical work? She must clearly be held to be incompetent, and the Midwives Board is right in declining to encourage a purely examination standard for admission to the register. It is essential, if the claims of the mothers are to be adequately considered, that the standard shall secure a thorough practical training for every midwife admitted to practice by the Board. Much more might be said, and many instances given of the dangers of the course favoured by the Local Government Board. But we venture to hope, that the Privy Council may reconsider the matter, and that the Midwives Board may be upheld in their determination, to maintain one standard for midwives and one standard only.

### The Care and Nursing of the Insane.

By Percy J. Baily, M.B., C.M.Edin., Medical Superintendent of Hanwell Asylum.

### II.—NURSING THE SICK.

(*Continued from page* 85.)

Ventilation must necessarily depend upon the movements of air. Now the movement of air may be brought about either by natural or artificial means. Hence we speak of ventilation as being either (*a*) natural, or (*b*) artificial.

(*a*) *Natural Ventilation.*—There are two physical laws which govern the conduct of all gases. These are: (1) All gases mix with one another by diffusion. Hence in large crowded places such as theatres churches, etc., the carbonic acid which is pouring out of the lungs of the individuals who compose the audience, etc., does not remain as a sort of canopy or envelope over their heads, but rapidly becomes diffused throughout the whole area of the enclosed space. (2) All gases when heated expand. In consequence of this law a given volume of cold air is heavier than an equal volume of heated air and hence hot air rises towards the ceiling, whereas cold air falls towards the floor. In Nature the production of winds depends chiefly upon this rising of heated air and the rushing in of colder air to take its place. The easiest and simplest way to ventilate a room is to take advantage of the winds by opening the windows and allowing the air to blow through (ventilation by perflation).

In an ordinary room or ward the means that we have of admitting fresh air and removing foul air are:—(1) The windows; (2) the chimney; (3) the door; (4) ventilating openings (if any). All these may serve in turn to remove the foul air (outlets) or to admit (except the chimney) fresh air (inlets). The door, however, must not be depended upon as an inlet since the air which would come through it (unless it be an outer door) would not be fresh air from the outside but air from the passage and other parts of the house, and therefore probably already partially polluted.

An immense volume of air passes up the chimney when a fire is burning in the grate, and this air, which, of course, comes from the room must be replaced by air which comes from the outside, which finds its way in through open windows (or the chinks around the sashes if they are closed), ventilating openings (if present), and the door. This is the reason why, in a sick-room, the fire should always be burning except in the hottest weather.

Theoretically, since heated, respired air rises towards the ceiling the outlets for the foul air should be in the upper part of the room, and where ventilating openings are provided those which are intended as outlets are always placed in this position either over the door or high up over the fireplace, the latter communicating with the chimney. Such openings are frequently guarded by valves or flaps so as only to permit the air to pass in the desired direction. Ventilating openings which are intended as inlets are generally arranged to open into the room, not on a level with the floor, where they would be likely to produce an uncomfortable current of cold air and give rise to cold feet in the occupants of the room, but at a height of about six feet from the floor. Such openings communicate with the external air by means of a tube, the outer opening being level with the floor (Tobin's tubes).

Windows open at the top rarely act as outlets, but serve almost constantly as inlets. Where there are windows on both sides of the room or ward these would generally act as inlets on the side from which the wind is blowing and as outlets on the other side.

Cold air is not so harmful as many people would seem to suppose, but draughts should certainly, if possible, always be avoided. This is best done by having several windows (all, in fact) always kept open a little way at the top. This is better and more effective than opening only one or two widely.

Every morning at least the windows of the sick-room or ward should be thrown open as widely as possible both at the top and bottom, when the weather will allow, for some minutes. During this time, in order that the patient may not feel the draught or cold, an extra blanket may be thrown over him. As the opening of the windows in this manner would tend (except in quite hot weather) to very much lower the temperature of the room, there are many cases, more especially of acute diseases, such as bronchitis, pneumonia, and the infectious fevers, where the procedure would be dangerous, but in nearly all cases of chronic disease good rather than harm is likely to result from it.

It must be remembered that the accumulation of steam on the windows inside in the form of drops of water is a sure sign of bad ventilation, and should never be seen.

During the whole of the daytime the windows of all dormitories and unoccupied sleeping apartments should be thrown wide open both at the top and bottom, and during the night the same should be done in all day rooms, passages, and other unoccupied spaces. Much may be done to prevent draughts by the use of screens.

(b) *Artificial Ventilation.*—When the air in rooms, wards, theatres, etc. is set in motion by some sort of machinery—usually revolving fans—the system of ventilation is said to be artificial. These revolving fans either force the fresh air into the room or ward or draw the foul air out. The former is known as the plenum system, the latter as the vacuum system. Both these systems of artificial ventilation require an elaborate arrangement of flues and ventilating shafts along which the currents of air move, and the necessary machinery for driving the revolving fans. When the plenum system is adopted the fresh air is generally made to travel over pipes heated by hot water or steam so as to warm it before it is thrown into the room, the foul air being allowed to find its way out through the ordinary openings or being drawn out by a series of extracting fans, in the latter case the two systems—plenum and vacuum—being combined.

*The Temperature of the Sick-room or Ward* should be kept as constant as possible, and in order to judge it there must be a thermometer which should hang on the wall as far as possible from both windows and fireplace. As a general rule about 60°

is the proper temperature, but in dealing with aged people and children, cases following surgical operation, and some pulmonary affections, it should be rather above this—from 65° to 70°. Phthisical patients (consumptives) may be submitted to very low temperatures without any fear of harm, as is frequently done in the open-air treatment of this disease—the only treatment which gives any real hope of cure. If the temperature of the room should fall below 60° the fire must be increased, but the windows are not to be closed.

(*To be continued.*)

### The Nurses' Clinic.

### THE DISTRICT NURSE AND THE PRESERVATION OF EYESIGHT AND HEARING.

A LARGE proportion of the cases of partial or complete blindness and deafness are caused by neglect of the simplest precautions during the first few weeks of a child's life; and it is no exaggeration to say that strict cleanliness during that period would prevent far more disease than the most skilled oculists and aurists can cure. Everyone recognises the terrible loss entailed by total blindness or complete deafness; but the weakening of the powers of eye or ear does so much to hinder mental and even physical development, casts such a shadow of gloom over childhood, and so pitiably impoverishes and embitters maturity, that any person with the smallest foresight can only look upon these diminished faculties as a slightly less misfortune than entire deprivation.

Considering the extreme and obvious fragility of infants, it seems strange that any mother should need to be taught that the younger a child is the more easily and irrecoverably it can be injured, that at five days old sight and hearing are more readily affected than at five months, and at five months than at five years; but nevertheless it is often at that low level that the nurse must begin her instruction. The second point to be remarked is that little but cleanliness, common sense, and affection are required in order to avert most of the evils with which the child is threatened.

Every morning when preparations are made to wash and dress an infant, six pieces of absorbent wool about the size of filberts and one larger piece should be laid on a square of mackintosh or clean paper. An egg-cup must be warmed with boiling water and then refilled with the addition of half a saltspoonful of powdered borax. When the water feels just warm to the tip of the finger, dip one of the pieces of wool into it, and wipe gently but firmly once across the child's eye, passing from the inner corner to the outer. The reason for washing in this direction is that only one eye may be in real need of attention, and if you wash from the outer corner inwards the sound eye may easily be infected by the unhealthy one. Nurses are sometimes told to wash towards the nose, because that is the way the watery currents on the surface of the eye flow; but these instructors forget how entirely deficient the baby is in bridge to its nose.

A fresh piece of wool must be taken for the other eye. Then, using a clean swab each time and never dipping a soiled piece into the egg-cup, both ears and both nostrils must be washed. The larger piece of wool must be dipped into a mixture of glycerine and borax, and the baby's mouth, gums, and tongue thoroughly washed. (For the prevention of thrush the mouth must be cleaned after each meal with soft rag dipped in warm water.) All the soiled wool should then be burnt. The nurse must be careful not to use her handkerchief nor to touch her face with her hands until she has disinfected them. Everything used must be scrupulously clean, and the mother will find it a great saving of time if she keeps egg-cup, borax, and wool in a box lined with clean paper and shut up in a drawer.

If, instead of being a healthy normal child, the infant, from neglect, has become one of those poor little creatures whose eyes discharge pus, whose eyelids stick together when it falls asleep, and whose eyelashes are pulled out when it wakes, a doctor must be consulted. The same nursing treatment will probably be ordered, but it will be applied every two hours night and day, and the eyelids must be well smeared with pure vaseline. The mother may truly be assured that the process does not hurt the child; if it cries, it is generally because it is not properly held and is afraid of falling, as can be seen by the clutching action of its hands. Even when the case had seemed almost hopeless, I have known successful results when this simple treatment has been persevered in. The mother must be warned never to poultice a child's eyes, and if one eye is healthy and one diseased, the sound eye must be carefully covered up while the other is being bathed. A young child must always be shielded from strong light, but if in this unhealthy state even ordinary daylight may be too dazzling for the inflamed eyes.

Care for eyesight must not end with infancy. Night and morning, and at least once during the day, young children's faces should be washed with clean soft water. and every trace of soap bathed carefully away. If at any time the eyes look inflamed they should be bathed with warm boracic lotion, and medical advice must be obtained as soon as possible. If grit should blow into the eye, the safest plan for untrained people is to bathe it gently with warm water, drop two drops of castor oil into it, put a soft pad and bandage over the eye to check involuntary movement, and persuade the child to lie down and go to sleep. If the grit does not disappear, or the pain continues, consult a doctor.

All mothers must be advised to observe their children's actions when reading, sewing, or trying to discern distant objects, and to take them to a doctor at once if there appears to be any abnormality. Many defects can be checked or cured if discovered early enough, which if neglected can only go from bad to worse. What is called "weak sight" is generally a sign of an anæmic condition, the power of vision varies from time to time, is now better, now worse, and the complaint will yield to very simple treatment, such as better food and more air. If, on the other hand, there is any structural defect causing short sight or squinting, carefully chosen spectacles should be regularly worn and changed every few months, if necessary. Many parents have a strong objection to disfigure "the pore little dear" with glasses, and sometimes very plain speaking is needed as to the relative value of being "a pretty appearing child" and a purblind adult incapable of earning the barest subsistence. Ventilation has a great effect on eyesight, and must be provided for, especially in the long winter evenings when the one living-room is filled with fumes of tobacco, cooking, and an ill-trimmed lamp burning the cheapest oil.

The days of infancy once past, the greatest dangers to the power of hearing arise from excessive colds, blows, picking at the ear with a sharp-pointed pencil or crochet-hook, and poverty of blood, resulting in abscesses. Any pain in the ear that does not quickly yield to warmth and rest must be looked on as a serious matter, and as the progress of diseases of the ear is extraordinarily rapid and only the earliest stages are hopeful, no time must be lost in obtaining competent advice.

### Incidents in a Nurse's Life.

MY FIRST PRIVATE PATIENT.

Who does not know the intense excitement which a nurse experiences on being sent to her first private case? The hospital days, where one is more or less under orders and discipline, are over, and the time has come when one has to stand alone and put into practice all the knowledge and skill which has been acquired under very different surroundings. I shall never forget the secret longings and ambitions with which I set forth. I had joined a Nursing Home in London, and it was a lovely day in summer when I drove up to the Home to begin my trial month. As I came straight from staying with friends at the seaside I was wearing private dress and not uniform, as I afterwards fervently wished I had.

I was shown into the matron's pretty little sitting-room, and to my astonishment her first exclamation was one of unmistakable vexation. She saw my surprise, and hastened to explain that she wanted to send me out *at once* to an urgent case, and was much distressed to find that I was not in uniform, because it would so much delay matters if I stopped to change. I told her that I could do so in a very few minutes; but she replied that I must go as I was, and take my apron, cap, and cuffs with me.

She said that she had given orders for my cab to be kept, and handed me my papers already written out and addressed—in fact, hustled me out of the house, and before I knew where I was, I was being driven swiftly away through the streets of London. I read and re-read the direction on the envelope: "Mrs. ——, Bond Street, Islington." I wondered whether it was Mr. or Mrs. —— who was ill, and what was the matter; for I had not been able to ask any questions in the hurry of my departure, and I pictured all sorts and conditions of houses and of welcomes, but none came anywhere near the truth. As the cab drew up at a public-house in a very dirty back street I was aghast; and if I had not seen "——, Licensed to Sell Wine and Spirits," placarded up I should have thought that I had come to the wrong place. I paid the cabman, and was sorry to see him quickly mount and drive off, for at least he seemed an old friend. Taking my courage in both hands and trying to look very prepossessed and quite at home, I marched boldly in and asked for the innkeeper by name. I stood in the passage waiting while a poor little down-at-heel servant-girl went to fetch him. I heard her state the fact that a young lady wanted to see him. As she said "Mr. ——" the announcement was greeted with roars of laughter. "A young lady, Jim, and the old un' not gone yet!" Again roars of laughter burst forth, and they told the maid to fetch the young lady in. My courage had almost deserted me by this time, and, trembling very much, I entered the room to find three men sitting round a table steadily drinking, whilst a fourth lay on the sofa already dead drunk. The fourth I found to be none other than Jim, the innkeeper. Strange to say, the sight of these men drinking whilst the wife lay dying upstairs—which fact I had gathered from their own remarks—made me so righteously angry that I no longer felt either nervous or frightened. I stated my business in a firm voice and said I wished to be taken to the sick woman at once. The men broke out in chorus: "Go on! you're not a nurse dressed up like a lady"—"Give us a kiss under that fine hat"—"I say, you're a pretty girl." I held my ground, and, turning away, told the little servant girl to take me upstairs at once to her mistress. I think I shall never forget the sight of that dirty, untidy room and the poor woman lying there in bed. I saw at once that she was dying, but I started to make her as comfortable as I could under the circumstances. There was a lot to do, and I made the little girl stay and help me, and felt quite repaid at the end of an hour when my patient feebly said "Thank God you came," and tried to squeeze my hand. The doctor arrived just as I had finished my work and looked with approval at the bright fire burning, the bed with its clean sheets, and at the other improvements which I had tried to make, but his face fell as he noted the chart and felt the patient's pulse. He ordered many things, first leeches to relieve the congested heart, and then poultices and a steam kettle, and as I followed him out of the room he said: "Nurse" (I had by this time donned my cap, apron, and cuffs, and was immensely relieved to be called "nurse" once more) "you have done wonders with the room and the patient, but it is all too late, I fear she can't live many hours." I begged him to speak to the husband downstairs and to try and get rid of the noisy drunken crew, for at times I could hardly hear myself speak for the shouting and singing below. He promised to do all in his power, and after a good deal of noise there was sudden quiet and I heard the little maid girl locking the door. It was now about 10 o'clock. The doctor said he would look in again later to see how the treatment had answered, but in an hour's time I saw that the end was very near and sent for him. The husband was so soundly asleep that I feared to waken him in his drunken state, and my poor patient had long ago become unconscious to all that was passing around her. The doctor came just as she passed away. I did all that remained to be done and then wondered where I could possibly stay till the morning. It was now about 12.30. I found that the bedroom where the dead woman lay was the only room free, the rest of the house being let to different sets of lodgers. Downstairs the drunken husband lay on the floor in one room, the kitchen was let to two young men, and I came to the conclusion that nothing but the bar was left uninhabited. So there the little maid and I sat till the dawn broke. You may be sure that I was very thankful when the daylight came and I could go away to the Home, leaving the husband still snoring loudly on the sitting-room floor. I was very much afraid the matron would say I ought to have waited to obtain the fees for the case, but I think when she had heard my tale she realised that I was best away, and it did not matter as the money was sent the next day in a dirty bit of paper for "The Matrin." I felt so pleased, too, when later on matron told me that the doctor had telephoned to her saying some nice things about the new nurse. I have been into many funny places since, both luxurious and poor, and met with much kindness and consideration in both, but I shall never get over the horror of that public-house nor forget the smell of stale liquor and dirty people which pervaded the bar that summer night.

### To Nurses.

We invite contributions from any of our readers, and shall be glad to pay for "Notes on News from the Nursing World," "Incidents in a Nurse's Life," or for articles describing nursing experiences at home or abroad dealing with any nursing question from an original point of view, according to length. The minimum payment is 5s. Contributions on topical subjects are specially welcome. Notices of appointments, letters, entertainments, presentations, and deaths are not paid for, but we are always glad to receive them. All rejected manuscripts are returned in due course, and all payments for manuscripts used are made as early as possible after the beginning of each quarter.

### The Army Nursing Service.

BY MR. ATHELSTAN RENDALL, M.P.

Some comment having been made in two nursing journals on my questions to Mr. Haldane on the conditions of the above service, I venture to address the following remarks to you.

If we regard a nurse as a woman highly trained in a profession second to none in the world, and necessarily of discreet age, it seems strange that in the twentieth century she should be treated as if unfit to look after herself. Years ago women were the legal chattels of their men-folk. The Army Nursing Board would apparently have them regulated and controlled in a similar manner by the public service.

The officer in the Army and Navy, and also the female Post Office clerk, has his or her appointed hours for being on and off duty. The public service rightfully demands that they shall be competent and punctual in the performance of their duties, if they misuse their off-duty hours their work is impaired and their continued employment or promotion is rightfully jeopardised. The *Journal of Nursing* writes: "If she (the nurse) is dancing till the small hours of the morning it is very improbable that she will be fit to take up her work next day at the usual hour." Very true; but has the medical officer no nerve and strength straining duties? Has he no need of being in the pink of condition for their performance? Is he to be allowed to dance till the small hours of the morning, and shall we rest assured that such is his wisdom, strength, and discretion that the steady hand and nice judgment that may be required of him a few hours after are certain to be there?

It may be said that he may be trusted to use his liberty in such a manner as not to abuse it or to injure his patients. Precisely the same reply can be made on behalf of the nurses. Nightly dances are not weekly or even monthly pleasures that professional women have either the chance of or the desire for. The same journal continues: "It is not desirable that any nurse should be dancing through the night with the medical officer under whose directions she performs her work," and speaks of "the due observance of the usual etiquette between their respective professions." Do doctors and nurses then belong to different social spheres? This information, afforded in a journal for nurses, will afford the nursing profession some little amazement. Nurses have been known to marry doctors more than once without any very disastrous social or other consequences to their husbands. Further, nurses at Netley play Badminton and lawn tennis with the doctors, go sailing with them, and take part in paper chases with them. During the South African war there was either no dancing restriction or it was relaxed. Nurses at Netley go to parties attended by doctors and there is no information at my disposal to prove that on these occasions the nurses have their meals in the kitchen. Nurses sometimes go for walks with doctors when off duty with, I believe, no injury to society or the due performance of their duties. If a cultivated woman ("lady" or "gentlewoman" if preferred) belonging to a noble profession cannot be trusted to behave herself as she should, or to so use her hard-earned leisure as not to injure her health, it appears to me that she ought to be turned out of the profession. As a matter of fact, the absurd and antiquated regulation that treats her as a child or a fool or (is this it?) a wily snapper-up of unconsidered trifles (such as doctors and officers) is one for which there is no honest justification. We treat men as men. It is quite time that we treated women as women. Independence is the only ground on which to build character.

I may perhaps say that I am disinterested in making these remarks because I regard dancing as a stupid "method of barbarism," all the more stupid because indulged in by civilised people in artificially lighted and badly ventilated rooms instead of, as in the case of genuine savages, in the sunshine and pure air.

This in no way detracts from the injustice of the present sex disability.

### Appointments.

Alfred Bevan Memorial Convalescent Home, Sandgate, Kent.—Miss Maud Fletcher has been appointed matron. She was trained at the Sunderland Infirmary, has since worked as Queen's district nurse at Bath, been private nurse at the London Association of Nurses, New Bond Street, W., and has been nurse-in-charge and matron's assistant at the City of London Freemen's Orphan School, Brixton, S.W.

Borough Fever Hospital, Bolton.—Miss Agnes Routledge has been appointed sister. She was trained at Clayton Hospital, Wakefield, and Monsall Fever Hospital, Manchester. She has since been staff nurse at the Hospital for Women and Children, Leeds, and charge nurse at the Infectious Diseases Hospital, Birkenhead.

Borough Sanatorium, Folkestone.—Miss Ethel M. Blaker has been appointed charge nurse. She was trained at the Hove Sanatorium, near Brighton, and St. George's Infirmary, Fulham Road, London. She has since been charge nurse of the Hove Sanatorium. She has also done private nursing.

Chorley Isolation Hospital.—Miss A. Delgano has been appointed charge nurse. She was trained at Middle Ward Hospital, Motherwell, where she has since been staff nurse. She has also been charge nurse at the Isolation Hospital, Martin Moor, Derbyshire.

Cottage Hospital, Copse Hill, Wimbledon.—Miss Margaret Morris has been appointed staff nurse. She was trained at the Gravesend General Hospital, and has since been night sister at Hereford General Hospital. She has also done private nursing.

Fort George.—Miss Anna Sinclair has been appointed Alexandra nurse. She was trained at the Glasgow Western Infirmary, and has since been assistant matron at Queen Victoria Hospital, Las Palmas; sister at the Deaconess Hospital, Edinburgh; sister-in-charge of a Glasgow Nursing Home; midwifery pupil, and midwife at the Hospital for Women, Brighton. She has also done fever, district, and private work. She holds the certificate of the Glasgow Western Infirmary, the Brighton certificate in midwifery, and the certificate of the Central Midwives Board.

Hackney Union Infirmary, Homerton.—Miss Nellie Maria Rance has been appointed assistant matron. She was trained at St. Marylebone Infirmary, and has since been sister and night superintendent at St. Pancras Infirmary. She has also been charge nurse at the North-Western Fever Hospital.

Jessop Hospital for Women, Sheffield.—Miss Rose Mathews has been appointed sister. She was trained at the County Hospital, Durham, where she afterwards became sister. She has since been outdoor nurse at the Infirmary, Berwick-on-Tweed.

Longton Cottage Hospital.—Miss Sarah E. Barlow has been appointed matron. She was trained at the Stanley Hospital, Liverpool, and has since been special nurse at the Jessop Hospital for Women, Sheffield, head nurse at the Longton Cottage Hospital, and matron of the Fleetwood Cottage Hospital.

Royal Boscombe and West Hants Hospital.—Miss E. M. McArthur has been appointed charge nurse. She was trained at Camberwell Infirmary, London, and has since been engaged on the private nursing staff at the Royal Infirmary, Preston.

St. Mary's Hospital, Paddington.—Miss J. Hills has been appointed night superintendent. She was trained at King's College Hospital, London, and has since been night sister and ward sister at the North-Eastern Hospital for Children, Hackney Road. She was subsequently holiday sister at St. George's Hospital, London.

The New Hospital for Women, Euston Road, London. Miss G. E. McNeil has been appointed matron. She was trained at St. Bartholomew's Hospital, London, and at the Dumfries and Galloway Infirmary. She has since been second assistant matron at Southwark Infirmary, East Dulwich, S.E., and assistant lady superintendent and matron at the Royal Infirmary, Liverpool.

### Presentations.

City of London Freemen's Orphans' School.—On Wednesday last week Miss Maud Fletcher was presented with a striking clock with inscription and a gold brooch by the officers and pupils of the City of London Freemen's Schools, Brixton, on the occasion of her leaving for the Alfred Bevan Memorial Convalescent Home, Sandgate, of which institution she has been appointed matron.

### Everybody's Opinion.

### THE C.M.B. EXAMINATION.

"Interested" writes: It is very gratifying to notice that all the pupils trained at the Maternity Department of the Cardiff Queen's Nurses' Institute passed the recent examination of the Central Midwives Board held at Bristol in October. This result must be especially gratifying to the Maternity Superintendent, as most of the pupils trained at the Home are without any previous nursing experience.

### FOUR MONTHS' TRAINED MIDWIVES.

Dr. A. B. Calder, of 652 Fulham Road, writes: I am well aware that the Central Midwives Board only insists on three months' training to qualify candidates for the C.M.B. examinations, but if the spirit of the Midwives Act is to be carried out as expressed in the sub-title, "to ensure the better training of midwives," great praise is due to those matrons who limit the three months' course to nurses who have had hospital training and experience. It seems to me that this question of the nurse being "trained" or "not trained" must be left to the discernment of the matron herself, and in any case a matron may conduct her training school on those lines which she considers most advantageous to her pupils. All the same I think it a pity that matrons of training schools should not be unanimous in insisting on four months' training for untrained nurses. Such unanimity would strengthen greatly the best interests of both matron and midwife. The fewer the three-months-trained midwives who are enrolled the better.

### THE REGISTRATION OF NURSING HOMES.

"Y." and "Z." write: We are so glad to find that the question of registration of Nursing Homes has again been taken up. So many nurses are caught at this time by the following advertisement: "Salaried nurses wanted at once for private nursing. Co-op. later." They little know that it equals salary during the winter months (*i.e.* £28 to £30 a year), while the matron receives their fees (two guineas per week). About the month of May, when things are getting slack, they are put on the co-op.; then when the busy season commences the above advertisement again appears, and those unfortunate nurses who have been patiently waiting for work are either asked to go, or things are made so uncomfortable for them that there is no choice left but to seek another "Home"! Many instances have come under our own notice where the nurse has asked for a testimonial and been refused, no reason being given. How, then, on seeking for an appointment is she to account for those months spent in the service of that matron?

### THE STANDARD OF NURSING.

"Nurse K. M. L." writes: I quite agree with "A Sister" in her letter in which she remarks about the advertisement. No fully-trained and properly-qualified nurse is at all likely to marry a man who is, or is to be, a porter. I understand a porter's duties are to attend doors, light gases, carry letters, etc., and they are usually old Army men or something of that sort, and if his wife is to take charge of the hospital, how vastly different their various duties will be! I have often remarked to my fellow-nurses about these advertisements, and wondered if anyone else noticed it. However, I am glad to see that someone else has. I have been trying to imagine some of the trained nurses I have met and worked under married to a porter, but the effect is so ludicrous I have given it up. Nurses do not move in the society of hall-porters.

### COAL FOR SICK-ROOM FIRES.

"E. C." writes: In "The Nurses' Clinic" last week mention is made of the method of wrapping coal in pieces of paper to prevent a noise disturbing the patient during the night. I attended a lecture on nursing last week given by a lady of considerable experience, and as her views quite coincide with my own I venture to send them to you. The lady in question said that she herself had never tried the method save on one occasion, when the results were nearly disastrous. The patient—a serious operation case for whom sleep was almost a matter of life and death—was dozing when the flare of the paper in which the coal was wrapped woke him in a state of considerable alarm. The nurse—owing doubtless to his weak condition—had considerable difficulty in persuading him that the house was not on fire, and inducing him to remain in bed. She further pointed out to the audience that with a housemaid's glove pieces of coal can be put on absolutely noiselessly. The only proviso was that the pieces be large enough and that no small coal which requires a shovel be used—at any rate during the night.

### MALE NURSE AND MEDICAL SUPERINTENDENT.

Nurse W. H. Hutton writes: With regard to my action against the medical superintendent of the Wandsworth Infirmary, I should be glad if you would allow me to state that I belonged to the 19th P.W.O. Hussars stationed at Bangalore in 1895, I was trained in nursing, first-aid and stretcher drill, and attended the usual course of lectures, after which I was trained in ward work—surgical, medical, and mental—under the lady superintendent of the Madras Presidency, Sister Leo Maxwell Muller, R.R.C. I obtained my certificate for nursing after passing my examination. After this I was employed in the General Hospital at Bangalore and again at Secunderabad as permanent nursing orderly, doing exactly the same work as a staff nurse at a civilian hospital for about five years. Later on I worked at the Moor River Hospital, Natal, South Africa, under Sir Frederick Treves and Sir William McCormack and others. Since my return I held private appointments until I obtained the post of assistant male nurse at the Wandsworth Infirmary, where I remained for eighteen months. I left with good testimonials, and Dr. Neal told me that he would be only too pleased to speak for me if I sent anyone to him. I am glad to say I am in a very comfortable place now.

BABY'S COMFORTER.

"Trained Nurse" writes: I was greatly interested in an article on "Rubber Teat and Jaw Deformity" in the *British Medical Journal*, October 20, by Dr. Tom F. Bedley, of Rangoon. In it the writer, after denouncing the present system of bottle feeding, passes on to the subject of the "Comforter," and remarks: "Apart from its mechanical effects upon the jaws and teeth, it introduces into the child's mouth dirt and micro-organisms, and causes an abnormal secretion of saliva detrimental to digestion." To my mind, it is the abuse and not the use of the "comforter" that is to be deplored. As an experienced and successful maternity nurse, I consider that whenever things can be satisfactorily managed without a "comforter" well and good. But I have found its use advisable in many instances, as when an infant (especially a male) cries unduly, or when the mother is delicate and it is imperative that she should have undisturbed nights. I simply place the "comforter" in the child's mouth after feeding, and remove it immediately it is asleep, which is usually in about ten minutes. When not in use I keep it soaking in boracic lotion.

THE DIFFICULTIES OF THE PRACTISING MIDWIFE.

"Close Observer" writes:—I wish to strengthen the statements made on "The Difficulties of the Practising Midwife." I worked on my own account, doing midwifery on district for five and a half years, and never found difficulty in obtaining work, but frequently had a case to give to any nurse-midwife coming to me to seek work. The medical men always treated me with the greatest consideration, and even when patients were unable to pay the usual midwifery fee at once the doctors never allowed me to be placed in any predicament respecting the mother or child, but willingly attended, leaving the fee to be discussed at a more convenient time. There are nurses who cannot get on with medical men, and these women also fail to agree with their fellow-nurses, the spirit of jealousy and direct antagonism one to another being a conspicuous—and pardon me for adding—an iniquitous trait in their character. Surely the strict discipline to which most have had to submit during the period of training should have knocked off these ugly corners, and created a spirit of humility and love for mankind, especially for members of the same noble profession, instead of fostering evil feeling one toward another. In all classes of trade wages are being sunk on account of increasing competition. Thrift and economy seem to be the exception rather than the rule. In fact, we live in an age of progressive extravagance, and unfortunately the trained nurse is tarred with this same disastrous brush. Judging from my own experience of nearly twelve years, I believe that there is a bright outlook in the near future for nurse-midwives, in spite of difficulties and supposed obstacles respecting adequate remuneration for time, brains, and money expended on training. At first, things always creep slowly, and nurses must be satisfied with a little. In due course the rural districts will afford ample opportunities for gaining a livelihood with the aid of a bicycle. Expenditure being less, the country nurse will be able to provide for old age as well as her sister in the town. It does not matter what branch of nursing a woman is engaged in the work is exhausting, both physically and mentally, and a nurse's working life is shorter than that of a woman engaged in any other profession or trade. Hence it is important that habits of thrift should be encouraged, a nurse daily learning to have more confidence in herself, treating everybody with respect and without partiality, so that a feeling of charity may be aroused and the nurse looked upon as a leader of her sex. Many of the "Gamps" have made, and are still making, a good living, with heaps of money to spare for the most expensive of beverages; and if "Sarah Gamp" can flourish, then be sure we can.

### Death in our Ranks.

We regret to record the death of Miss Fanny Newbold, who was a member of the staff of the Auxiliary Nurses' Society and a resident at the Nurses' Hostel, Francis Street, for many years. A floral cross has been subscribed for and sent as a token of regard by her fellow residents at the hostel.

### The Nurses' Bookshelf.

Practical Text-book of Midwifery for Nurses. By Robert Jardine, M.D. (Edin.), M.R.C.S. (Eng.), F.R.P. and S. (Glasg.), F.R.S. (Edin.). Third edition, with forty-nine illustrations. (London: Henry Kimpton, 13 Furnival Street, Holborn, E.C. Glasgow: 40 to 42 University Avenue. 1906. Price 5s. net. Pp. 276. Size, 7½ inches by 5¼ inches.)

The author's name and the fact of a third edition having been called for go far to make a *prima facie* case for the value of such a work as this for maternity nurses. The book is based on a series of lectures delivered to the nurses in the Glasgow Maternity Hospital. After a short introduction dealing with general principles, the author commences with a brief survey of the body cavities, systems, organs, and their functions, and this we consider an admirable prelude to a work, however simple, for midwives. The bony pelvis is next described and its diameters; the planes and the axis of the pelvis are also explained. The third chapter gives a concise account of the female organs of generation. We note in fig. 5, page 23, that the illustrations are not marked "A" and "B," according to the description given below the diagrams. Next follow chapters on the changes in the uterus during pregnancy (in which the formation of the deciduæ and placenta are explained), and the symptoms and signs of pregnancy. An important chapter, which should be of much value to the midwife, is that dealing with antiseptics and asepsis. The chapter dealing with the management of labour contains some useful figures illustrating the methods employed for the external examination (palpation of the abdomen), and the importance of this part of the examination is rightly insisted on. We recognise the conciseness and accuracy of the text, and we are of opinion that the book admirably fulfils the objects for which it has been compiled.

### Where to Go.

Sale of Patients' Work.—British Home and Hospital for Incurables, Wednesday and Thursday, November 28 and 29, at 3 p.m.

Hospital and Home for Incurable Children, "Northcourt" Swiss Cottage, N.W.—Saturday, December 1, 11 to 1 and 2 to 6. Pound Day.

Royal National Orthopædic Hospital, Great Portland Street, W.—Thursday, December 6, and Friday, December 7, 2.30 p.m. Sale of work.

### Novelties for Nurses.

(By our Shopping Correspondent.)

THE "SISTER MARY" CLOAK.

The Hospital Contractors and Nurses' Outfitting Association, Stockport, write to state that the price of the "Sister Mary" cloak, noticed, with other articles in our issue of last week, is 22s. and not 25s. That is to say, the price of the plain "Sister Mary" cloak with caps is 22s.; the hood is 2s. 6d. extra, and facing with good quality merve silk is 2s. 6d. extra—25s. together.

### A Book and its Story.

### A PARENT'S PURSUIT.*

There is no lack of incident in Miss May Crommelin's new novel, which carries the reader from an English village where the heroine, Phœbe Garland, lives as the adopted daughter of Mr. and Mrs. Barker, tenants of the White Farm, to Northern Italy and other European places of resort. The girl acts as maid and companion to her god-mother, Miss Cunningham, lady of the demesne of King's Hoo, who, vivacious and middle-aged, goes in search of the distraction which life in Continental hotels provides for those in search of it. Phœbe, having lived with her relatives from childhood to womanhood, has but a dim recollection of any other home. In the absence of the former owner King's Hoo had been left in charge of her mother as caretaker when Phœbe was a mere child. In the indistinct outline which remains to her of that time the figures of a sad-eyed mother, and of a father who paid surreptitious visits, and disappeared as mysteriously as he came, alone stand out. The White Farm and its honest, kindly occupiers hold in Phœbe's heart the place of home and parents. When the story opens Miss Cunningham has broached the subject of Phœbe's coming to her as maid. Her uncle and aunt decline to discuss the matter. They have more ambitious views for their niece. A tacit understanding exists that some day she is to marry her cousin Edgar, who to them takes the place of a son, as Phœbe does of a daughter. But their benevolent prospects are cut short by an accident which ends fatally. They had driven into the neighbouring town to see a lawyer with regard to a new will which Mr. Barker had made. They do not return alive. By some mishap they are found in a pond with the cart overturned on them.

Previous to the catastrophe John Barker, a young Canadian—whose father, a brother of Mr. Barker's, had settled in Canada and not been heard of for years—appeared at the White Farm. He is a typical son of the new country—intelligent, virile, good-looking, and in strong contrast to Edgar Morris, who lacks the outspoken directness of thought and action cōnspicuous in the Canadian. He at once attracts the affection of his English uncle and excites enmity in the mind of Morris. Morris has philandered with Phœbe and done his best to make her devoted to him. She, in return, has mixed feelings of love for and annoyance towards him. After the death of the uncle and aunt Phœbe goes, very heart-broken, to live with Miss Cunningham. For safe custody before leaving home on the day of his death Mr. Barker had given his first will into the Canadian's keeping. The new will, which had been made in his favour as next of kin, required some slight alteration, and Mr. Barker was known to have had it with him after the visit to the lawyer. He had made the necessary alteration and witnessed Mr. Barker's signature. Up to the day of the funeral, although an ample reward has been offered, no trace of the new will could be found.

At the funeral the lawyer announces that, no will having been found, Mr. Barker's possessions will go to the next of kin. Then Barker, the Canadian nephew, produces the old will left in his charge by his late uncle. By this Edgar Morris comes into the possession of the White Farm lease after the death of Mrs. Barker and of everything left to her. Phœbe takes certain furniture and family relics. These were left with the unwritten understanding between the uncle and Morris that, as Morris's wife, she would be well provided for. But what actually occurred is explained by the following passage. It is the evening of the funeral day. Phœbe and Edgar Morris are alone. "Is there anything I can do for you?" he asked. Trembling, Phœbe rises, steadies herself against the table, and, addressing him as the master of the house, asks when he wishes her to leave the old home. "Oh, well," Edgar turned his back now, pulling the leaves out of Phœbe's pet fuchsia on the window-ledge. "I should be sorry to hurry you. Still, the sooner all gets on its new footing the better. . . . This is Thursday. Would next Monday do you?" "Yes." Phœbe presses her hand to her side, but Morris disregards her. He continues, "Very well, then, you can have the cart to move your furniture, as I suppose you'll go to the village." "I don't know." Phœbe's voice gives way and she breaks down entirely. Morris strides off and Phœbe is left alone in her sorrow. But Miss Cunningham hears of her distress, and hastens to console her, and in reply to Phœbe's declaration that she is homeless and heartbroken she tells her that a good home is waiting for her at King's Hoo if she will come there as her god-mother's "dear little companion and faithful maid and friend." So Phœbe departs at once and begins a new chapter in her life. The furniture and pictures that were left to her are sent after her and placed in the room that she is to occupy at King's Hoo. At a loss to understand the change in Edgar Morris's demeanour, her mind travels back to a conversation she had with Mrs. Barker one day before the fatal accident in reference to his uncertain treatment of her. She had asked her aunt if she thought it had any connection with her father, and if the knowledge of his being a professional thief would be likely to account for it. Mrs. Barker had been startled by the inquiry, horror stricken that Phœbe could be aware of the fact. She replies that Mr. Barker had thought it right that Morris should know, when, two years previous to this, he had been made steward and had been treated as one of the family. But it had made no difference then. Phœbe continues: "At first he would not care. Lately he may have begun to think. Mrs. Barker tries to console her by saying that no one knows of this except themselves. Her father's name even is unknown to them, and Phœbe was to them as a daughter taking after her mother's family—the Garlands—although she had had the misfortune to marry a scapegrace of good family with bad morals. When Phœbe is installed at King's Hoo, piece by piece the past comes back to her. She remembers a secret passage, by which her mother escaped with her in her arms to the White Farm when she was living at King's Hoo as caretaker. They were flying from her father who had paid a surprise visit to her mother, bent on robbing the mansion. Later, Phœbe is the victim of more than one encounter with the gentleman burglar, her father, alone, at first, and on other occasions with accomplices. She insists upon Miss Cunningham having a duplicate made of a very valuable pearl necklace, and other jewels, in consequence. The pursuit of his daughter by the felon father makes poor Phœbe's life one of constant torment; at every place he appears in a new disguise, and very exciting episodes occur in consequence. In fact, no recent novel, and certainly not one with so misleading a title as "Phœbe of the White Farm," contains more plots and hair-breadth escapes, nor more vivacious characters. Whether at home or abroad, the scenes and people described are vividly portrayed, and the adventures of Phœbe, ending, in spite of her unfortunate position, in well-earned happiness, make an exciting and admirably told tale.

* "Phœbe of the White Farm." By May Crommelin. (John Long. 6s.)

### Notes and Queries.

**REGULATIONS.**

**The Editor is always willing to answer in his olumn, without any fee, all reasonable questions, as soon as possible.**

**But the following rules must be carefully observed.**

1. **Every communication must be accompanied by the name and address of the writer.**
2. **The question must always bear upon nursing, directly or indirectly.**

**If an answer is required by letter a fee of half-a-crown must be enclosed with the note containing the inquiry.**

*Queen Alexandra's Nursing Service.*

(101) Will you tell me the names of the committee of Queen Alexandra's Imperial Military Nursing Service?—*Mona.*

Write to the Secretary of the Service, 68 Victoria Street, S.W.

*Mental Case.*

(102) Do you know of an institution which would receive a slight mental case able to help and pay very small sum?—*Hopeful.*

You had better advertise. We fear we cannot help you.

*Surgical Nursing.*

(103) Can you tell me where I can get surgical training in the summer, to brush up my knowledge?—*Nurse M.*

Possibly you might be admitted to your former training school. We presume that you do not wish to pay. Therefore, if you fail with your own school, it would be better to advertise.

*London County Council Schools.*

(104) How can I get a post in the London County Council's schools at nurse?—*Alice.*

Write to the London County Council, Spring Gardens, W.C.

*Central Midwives Board.*

(105) Can you tell me where to apply about the Central Midwives Board, and if I get the certificate can I practise as a midwife?—*Bristol.*

First get "How to Become a Midwife," from the Scientific Press, 29 Southampton Street, Strand, W.C., and then write to the Secretary, Central Midwives Board, Caxton House, Westminster, S.W. You can practise if you get a certificate.

*Canada.*

(106) Can you tell me if there is any opening for nurses in Canada?—*Edinburgh.*

No, because they have excellent training schools, and your qualifications are not sufficiently high to venture independently. You must know at least one medical man in Canada who will help you, or it is useless to venture without three full years' general training.

*"Asylum News."*

(107) Can you tell me the address of the "Asylum News"? *Mental Nurse.*

"Asylum News," 8 King Street, Richmond, Surrey.

*New Zealand.*

(108) I am going to New Zealand. If I train in a hospital there, shall I have the same standing as if I trained in Great Britain?—*Maori.*

You will find it wiser to train in New Zealand if you wish ultimately to take up private nursing there; and also you will stand a better chance of securing a hospital appointment.

*Nurses' Uniform.*

(109) Is it right that anyone should wear nurses' uniform when selling at a bazaar?—*Inquirer.*

Unless the wearer is a *bona fide* nurse, such a proceeding is in bad taste, but it is not illegal.

*Australia.*

(110) Can you tell me of any lunatic asylums in Australia?—*Shrewsbury.*

There is a long list in Burdett's Hospitals and Charities, price 6s. 4d. post free, Scientific Press, 28 Southampton Street, Strand.

**Handbooks for Nurses.**

Post Free.

"How to Become a Nurse: How and Where to Train." 2s. 4d.
"Nursing: its Theory and Practice." (Lewis.) ... 3s. 6d.
"Nurses' Pronouncing Dictionary of Medical Terms." 2s. 6d.
"Complete Handbook of Midwifery." (Watson.) ... 6s. 4d.
"Preparation for Operation in Private Houses." ... 0s. 6d.

Of all booksellers or of The Scientific Press, Limited, 28 & 29 Southampton Street, Strand, London, W.C.

### For Reading to the Sick.

### THE CHRISTIAN WARRIOR.

FIGHTING alone to-night,
With not even a stander-by
To cheer me on in the fight,
Or to hear me when I cry:
Only the Lord can hear,
Only the Lord can see,
The struggle within how dark and drear
Though quiet the outside be.

Lord, I would fain be still,
And quiet behind my shield,
But make me to know Thy will,
For fear I should ever yield;
O Lord, Thou hidest Thy face,
And the battle clouds prevail;
Oh grant me Thy sweet grace
That I may not utterly fail.

Fighting alone to-night,
With what a beating heart!
Lord Jesus, in the fight,
Oh! stand Thou not apart.

*Fourteenth Century Hymn.*

Now we can be braced to strength, as we draw into us the grace and power of God. Now we can have the inward peace of heart and mind, which faith receives and holds, even in the midst of trouble. The fiercest onslaught of the prince of the powers of the air need not move us in our place, if we are of those to whom God gives strength. While we seek to Him in faith, in the ways of His will, that strength comes ever freshly. The break-up of what is all the world to us will not rob us of the peace of God, if we know ourselves to be His care. It may be that as yet we can hardly trace clear signs of the Divine gentleness and love, and faith has ever to seek new strength. But after the storm and unrest comes the peace of new and richer life. All hope shall be fulfilled in the peace of God in the holy land.

Christ is our righteousness and our peace. He gives peace indeed, by dwelling in us and making His righteousness ours. The old nature does not yet give way at once and wholly. So our mind and heart are not always, or quite at rest, kept by the peace of God. But God sets us free from the chafing bonds of sin. He turns away His wrath, and turns us towards Him in holy fear and trust, to yield ourselves as servants of righteousness unto holiness. And as there is rest in work for God, so there is peace in war for God. There is peace with God when we submit ourselves to the righteousness of God, knowing His mercy, sure of His truth. When righteousness is fulfilled in us, peace shall be ours in full.

God's loving-kindness is the same, even though only faith can know of it. We trust Him to whom darkness and light are both alike, and whom they both serve. We would wish always to hear His voice speak cheering words; but other tones may at times be more what we need. Does He cloud over the world's brightness, and withdraw His consolations, that we may prize His loving-kindness, not only the tokens of it? Has He some work for us to do, which we can do best in the darkness? Is there something to be done in us, which grace can work best, when we have lost trust in self, and the strain on faith is hard? We wait for the morning, sure that we shall hear God's loving-kindness, and know that it has been the same when we could not hear it.—*Anon.*

